# The Neglected Arboviral Infections in Mainland China

**DOI:** 10.1371/journal.pntd.0000624

**Published:** 2010-04-27

**Authors:** Xiaoyan Gao, Roger Nasci, Guodong Liang

**Affiliations:** 1 State Key Laboratory for Infectious Disease Control and Prevention, Institute for Viral Disease Control and Prevention, Chinese Center for Diseases Control and Prevention, Beijing, China; 2 Centers for Disease Control and Prevention, Fort Collins, Colorado, United States of America; London School of Hygiene & Tropical Medicine, United Kingdom

## Abstract

The major arboviral diseases in mainland China include Japanese encephalitis, dengue fever, Crimean-Congo hemorrhagic fever (also known as Xinjiang hemorrhagic fever), and tick-borne encephalitis. These and other newly found arbovirus infections due to Banna virus and Tahyna virus contribute to a large and relatively neglected disease burden in China. Here we briefly review the literature regarding these arboviral infections in mainland China with emphasis on their epidemiology, primary vectors, phylogenetic associations, and the prevention programs associated with these agents in China.

## Introduction

Arboviruses (arthropod-borne viruses) are maintained in nature in cycles involving hematophagous arthropod vectors and susceptible vertebrate hosts [1]. At present, more than 550 arboviruses have been identified, among which are more than 130 virus species that can cause disease in susceptible vertebrate hosts [2]. Japanese encephalitis virus (JEV), dengue virus (DENV), Crimean-Congo hemorrhagic fever virus (CCHFV) (also known as Xinjiang hemorrhagic fever virus, XHFV), and tick-borne encephalitis virus (TBEV) are the four principal arboviruses of public health importance in mainland China at present [3]. There is a growing body of evidence indicating that other arboviruses are present and causing human infections and diseases in China. In this review, we summarize relevant information available in the Chinese scientific literature, highlight the current situation of arboviral infections in mainland China, and describe current practices and recommendations regarding surveillance and prevention measures.

## Japanese Encephalitis

**Figure 1:**
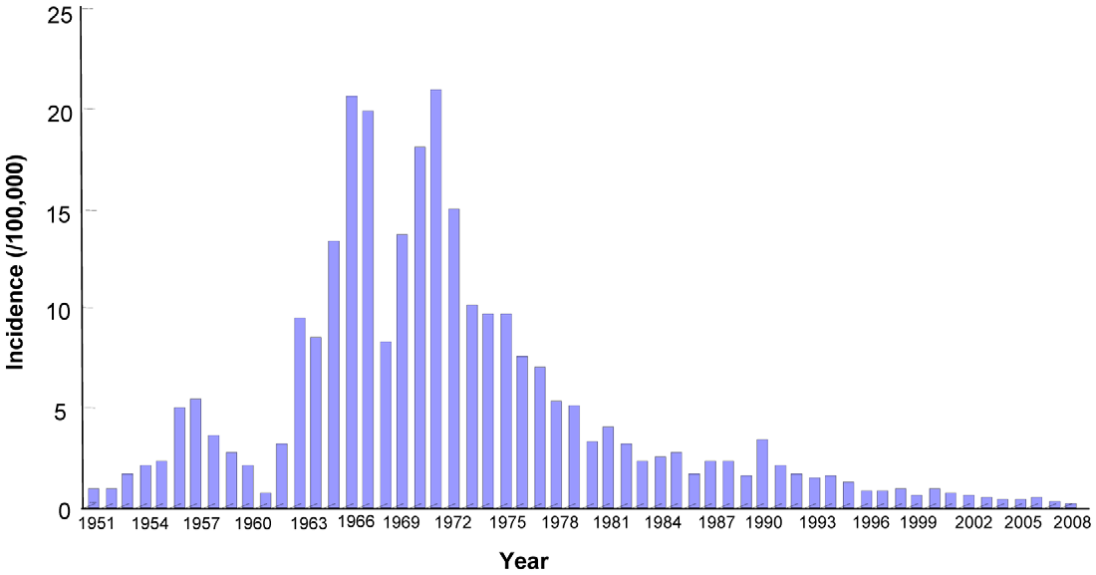
JE incidence from 1951–2008 in mainland China.

Japanese encephalitis (JE) is arguably the world's single most important acute viral encephalitis accounting for 30,000–50,000 cases and 10,000–15,000 deaths each year, with survivors often experiencing irreversible neurological damage [4], [5], [6]. By some estimates, the number of cases caused by JEV may be as high as 175,000 each year [7]. JEV is a mosquito-transmitted flavivirus that is concentrated in China, India, and Southeast Asia, where it is the leading cause of viral neurologic disease with an incidence exceeding that of herpes simplex virus [8]. The virus is maintained in a cycle involving birds and *Culex* mosquitoes. In Asia, an epizootic cycle involving domestic pigs and *Culex tritaeniorhynchus* mosquitoes is associated with high incidence of human disease in rural areas [9]. Historically, JE prevalence has been high in China, where major outbreaks occurred in 1966 and 1971 with reported disease incidence of >15/100,000 and 20.92/100,000, respectively [10]. After the nationwide vaccination program initiated in the 1970s, the number of reported cases dramatically decreased, with disease incidence declining from 20.92/100,000 in 1971 to 0.23/100,000 in 2008 (Figure 1) [11].

### Virus and Vectors

Nucleotide sequencing of the JEV envelope protein genes has identified five genotypes (GI, GII, GIII, GIV, and GV) [12]. Of these, GI and GIII of JEV circulate in China. GIII was widespread before 2000 while GI has been isolated from Yunnan, Shanghai, Liaoning, Sichuan, Henan, and Gansu provinces in recent years [13]. Both GI and GIII of JEV were detected from cerebrospinal fluids (CSF) of patients and mosquitoes simultaneously during a JE outbreak in Yuncheng City, Shanxi province in 2006 [14]. *Culex tritaeniorhynchus* is the primary vector of JEV in China, though the virus has been isolated from more than 20 other mosquito species. JEV also has been isolated from *Culicoides* and fruit bats in China [15], [16].

### Epidemiology

**Figure 2:**
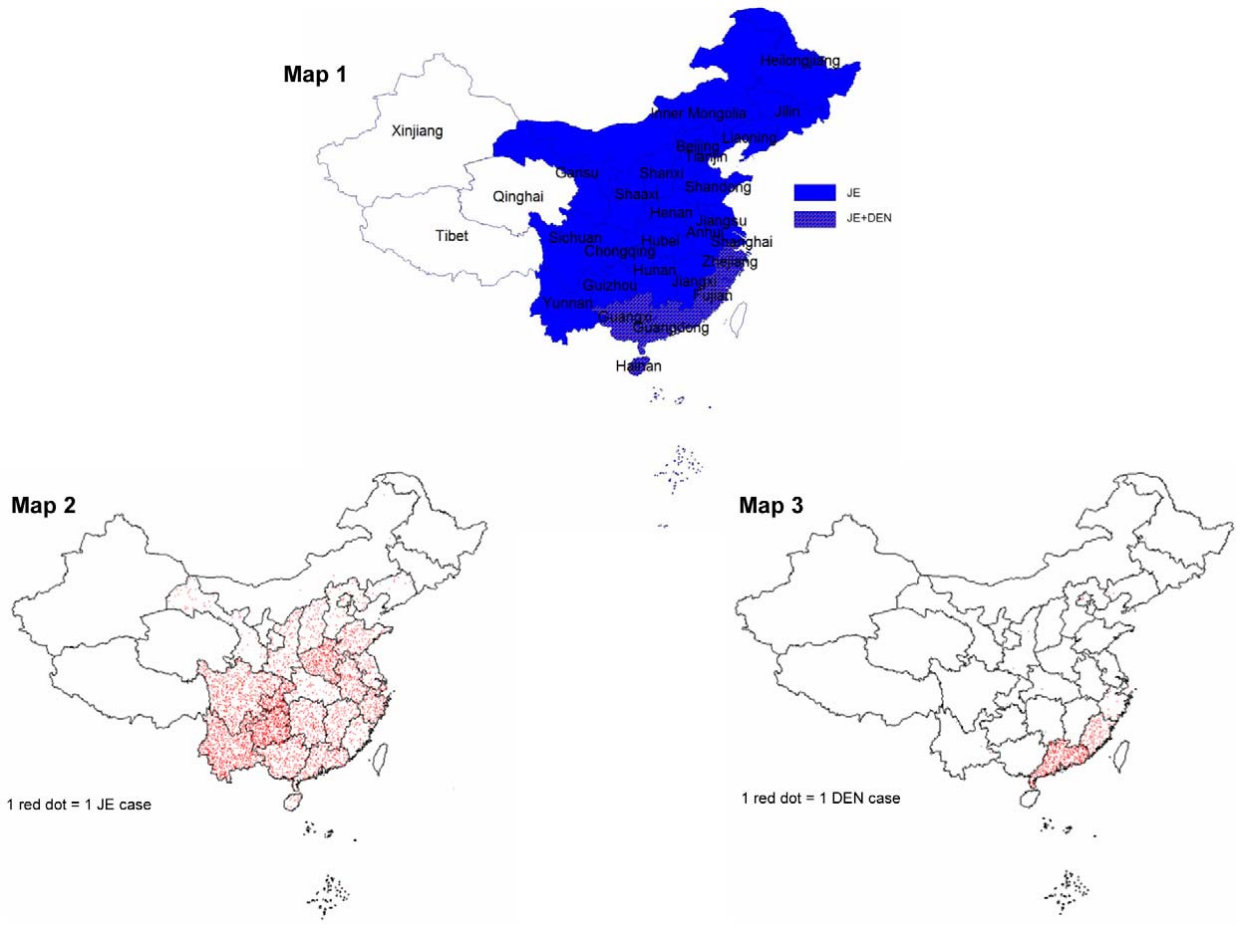
Distribution of mosquito-borne diseases in mainland China. Map 1 shows the distribution of JE and DEN. Map 2 shows the case distribution of JE in 2007. Map 3 shows the case distribution of DEN in 2007.

Human JE cases have been reported from all provinces with the exception of Xinjiang Uygur Autonomous Region (hereinafter referred to as Xinjiang), Tibet Autonomous Region (hereinafter referred to as Tibet), and Qinghai province in the western regions of China (Figure 2). JE epidemic areas can be divided into categories of high, medium, and low prevalence. Using case reports from 1998–2002, high prevalence areas include Shaanxi province, Chongqing municipality in Sichuan province, Sichuan province, Guizhou province, Henan province, and Yunnan province where JE incidence is over 1/100,000. JE medium prevalence areas include Anhui province, Hubei province, Hunan province, Jiangxi province, and Guangxi province where incidence is 0.5–1/100,000. In low prevalence areas such as Gansu province, Jiangsu province, Shandong province, Fujian province, Guangdong province, and Zhejiang province, the incidence is below 0.5/100,000. From historical and current data, it appears that high prevalence areas have shifted from eastern coastal areas to the central and western regions in the last 50 years [10]. Since 2000, the number of JE cases reported in China has decreased every year, with the exception of 2006 when the number of cases increased approximately 50% over the previous year.

JE cases occur during every month of the year in mainland China, while cases reported in winter are only from southern provinces. Generally, case reports begin to increase in May, peak in July and August, and decrease in September [17], [18]. During 2004–2006, the period from June to September accounted for 94.9%–98.1% of all cases within the year while July to August accounted for 78.9%–89.2% of all cases reported [17]. All age groups can be affected by JEV, but children account for most cases. During most years, children under 15 years of age account for 85.9%–90.6% of reported JE cases [10], [17]. However, an exception to this pattern occurred during the JE outbreak in Yuncheng city in 2006 when more than 86% of the patients were >30 years of age, with only 10% of patients <7 years of age [10]. Males are infected more frequently than females with a ratio 1.37–1.60∶1 probably because of more outdoor activity by males [18].

### Prevention and Recommendations

An inactivated JE vaccine derived from the P3 strain of JEV was developed in 1968 and was provided to populations at high risk in China [19]. In 1988 a live attenuated vaccine (based on the SA 14-14-2 strain) was licensed for use in China after studies had demonstrated its safety and high efficacy [20]. Currently, both vaccines are used nationwide and the vaccine program is largely credited with the reductions in cases that have been observed [21]. In 2007 the Chinese government announced its intention to include JE vaccine in the expanded project of immunization in order to further enhance the prevention and control of JE. Although great success has been achieved by the vaccination program, an enhanced JE surveillance system, including investigation of infection rates in vectors, should be implemented to better describe the burden of disease and risk factors for JE infection in different areas of the country. In addition, JE vaccination rates and immune responses following vaccination warrant further investigation.

## Dengue

Dengue (DEN) is caused by four serotypes of DENV that occur in over 100 countries and threaten the health of more than 2.5 billion people in urban, peri-urban, and rural areas of the tropics and subtropics [22]. DENV can cause a spectrum of illness ranging from dengue fever to the more serious dengue hemorrhagic fever/dengue shock syndrome (DHF/DSS) [23]. Worldwide, the virus causes about 50 to 100 million dengue infections each year, all of which may not present clinically as dengue fever. The estimates of DHF/DSS are close to 500,000 cases [24]. In China, dengue epidemics have occurred mainly in the southern regions of the country, primarily in Guangxi province, Guangdong province, Fujian province, Zhejiang province, and Hainan province (Figure 2).

### Virus and Vectors

All four serotypes of DENV have caused outbreaks in China. Large outbreaks were caused by DENV-4 in 1978 in Guangdong province, DENV-1 and DENV-3 in 1980 in Hainan province, and DENV-2 in 1986 in Hainan province [25], [26], [27].

The mosquito species *Aedes aegypti* and *Aedes albopictus* are the primary vectors of DENV in China. *Aedes aegypti* is the vector in coastal areas and is mainly distributed in Hainan province and found sporadically in Guangdong province and Guangxi province [28]. *Aedes albopictus* is the vector in inland regions and is widespread in mainland China. This species is distributed from Liaoning province in the north to Shaanxi province in the northwest and from Tibet in the southwest to the southern reaches of China beyond the Yangtze River [29]. In addition to detecting virus in the mosquito vectors, a study reported that DENV RNA has been detected using RT-PCR in the brain tissue of *Rousettus leschenaultia*, a fruit bat collected in Hainan province [30]. Moreover, antibodies to DENV were detected in *Rousettus leschenaultia* collected in Yunnan province during a study of dengue fever in the region [31].

### Epidemiology

In the early 1940s, DENV was epidemic on the southeastern coast of China and the middle and lower reaches of the Yangtze River [32]. There were no reported cases of DEN in China from 1946 to 1978. The reason for this phenomenon is unclear. In 1978, an epidemic caused by DENV-4 occurred in Foshan City of Guangdong province [25]. The outbreak affected seven neighboring counties, lasted 8 months, and resulted in 22,122 cases and 14 deaths. A dengue outbreak involving 13 cities and counties occurred in Hainan province in 1980, during which DENV-3 was isolated from acute-phase sera and adult *Ae. aegypti* [26]. This outbreak caused 437,468 cases and 64 deaths [33]. In 1985–1986, DHF was reported in Hainan province. This outbreak was caused by DENV-2 and produced considerable morbidity and high mortality, with 113,589 cases and 289 deaths [27], [33].

**Figure 3:**
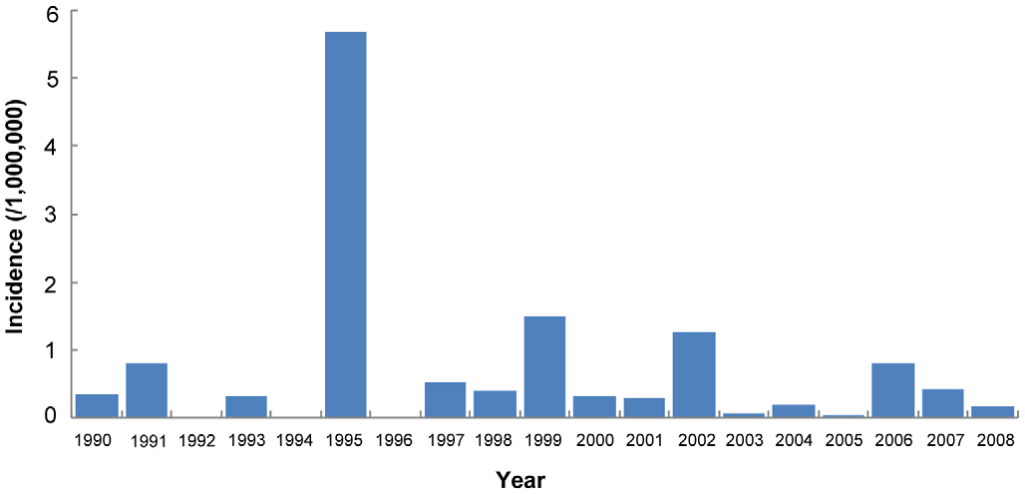
Dengue incidence from 1990–2008 in mainland China.

Since the 1990s, dengue epidemics have frequently occurred in Guangdong province, Guangxi province, and Fujian province. The outbreaks of dengue fever in China usually resulted from the introduction of the virus by infected travelers and refugees from various areas of southeastern Asia where dengue is endemic. For example, an outbreak of DENV-1 in Zhejiang province in 2004 was associated with a traveler from Thailand [34]. This outbreak caused a total of 82 reported cases. Epidemiological investigation of cases reported from Guangdong showed that cases reported from 1990 to 2006 were mostly imported or occurred in local epidemics initiated by imported cases [35], [36]. Nationwide in China, the number of reported cases has varied considerably, ranging from 40 in 2005 to 1,044 in 2006 with the incidence ranging as high as 5.7/1,000,000 over the period from 1990–2008 (Figure 3).

Though Yunnan province is adjacent to Vietnam, Thailand, and Myanmar where DEN is endemic, *Aedes albopictus* is relatively common in the southwestern part of the province, and DENV has been isolated from this species in the areas [37], [38], there have been relatively few DEN cases reported from Yunnan. However, increasing travel and commerce between Yunnan and the neighboring dengue epidemic countries increases the likelihood of imported dengue, which could become an important factor influencing dengue epidemiology in Yunnan in the future.

The epidemic season for DENV occurs mainly in the rainy, hot summer and autumn, which correlates with the seasonal periodicity of the vectors. Most cases are reported from March to November with a peak in July to September, while Hainan province has cases throughout the year with a peak in July to October [39]. Statistical analysis of incidence rates of age groups in different years showed that, in general, the highest incidence was in the 10- to 39-year-old group [40].

### Prevention and Recommendations

In the absence of an effective vaccine against DENV, vector management is the only prevention tool available. In China, vector control includes programs in community participation and health education to reduce mosquito breeding in household water containers. At present, a vector surveillance system has only been established in Guangxi province, Guangdong province, Fujian province, Yunnan province, and Hainan province. Vector-based surveillance should be established nationwide to determine the distribution of competent vector species and to identify areas that may be at risk if mosquito distributions shift in response to climate change. In view of the current status of a large number of imported cases, it is crucial to provide health education targeted at the high risk groups including travelers from dengue epidemic countries in order to prevent importation of dengue fever. In addition, considering the increased travel between Southeast Asia and China, more surveillance is necessary to monitor DEN epidemiology in the region.

## Crimean-Congo Hemorrhagic Fever

CCHFV is a tick-borne virus in the genus *Nairovirus*, family *Bunyaviridae* that produces a severe hemorrhagic fever and a potentially fatal outcome [41]. CCHFV has the most extensive geographic distribution of the medically important tick-borne viral diseases and the CCHFV infection has been described in parts of Africa, the Middle East, Eastern Europe, and Asia [41]. CCHFV causes severe disease in human beings with a reported mortality rate of 3%–30% [42]. Global disease burden is difficult to determine, however the incidence of sporadic cases and outbreaks of Crimean-Congo hemorrhagic fever (CCHF) is reported to have increased across the endemic region during the past decade [43]. The apparent increase in incidence possibly reflects both greater human exposure to infected ticks and more widespread recognition of the disease by health care workers [44]. In China, CCHF was first described in Bachu County along the Tarim river basin in Xinjiang in 1965. CCHFV was first isolated in 1966 in Xinjiang from the blood of patients, organs from autopsy, and the tick *Hyalomma asiaticum* [45], [46]. Based on the site of isolation in China, CCHF and CCHFV are referred to as XHF and XHFV in the Chinese literature [45].

### Virus and Vectors

Phylogenetic analysis of the S-RNA sequence showed that strains isolated from Xinjiang form a separate branch with isolates from its border regions (Tajikistan, Uzbekistan, and Kazakhstan). This suggests that CCHFV has developed a unique and possibly isolated focus of transmission in Xinjiang and its border regions [47]. The latest sequence information of CCHFV from China was obtained in a tick-borne arbovirus investigation in 2004 [48]. The phylogenetic analysis of partial L gene sequence of CCHFV showed CCHFV isolated in Xinjiang in 2004 has a distant evolutionary relationship to an African strain of CCHFV but a close relationship to central Asian strains from Pakistan and Tajikistan [48].

*Hyalomma asiaticum kozlovi*, a subspecies of *Hyalomma asiaticum*, is the main vector of CCHFV in China. The most active season for adult ticks is from early April to early May, which coincides with the epidemic season of CCHF and is also the busiest season for work in the pastures in this region. The virus can be transmitted to humans by the bite of infected ticks or by direct contact with blood and tissues from viremic livestock or patients. For example, there has been one report of a possible horizontal transmission from a mother to her child in Xinjiang [49]. So the latter route of transmission indicates that preventive measures should be used by farmers working with sick livestock as well as by caregivers to reduce the risk of human-to-human transmission.

### Epidemiology

**Figure 4:**
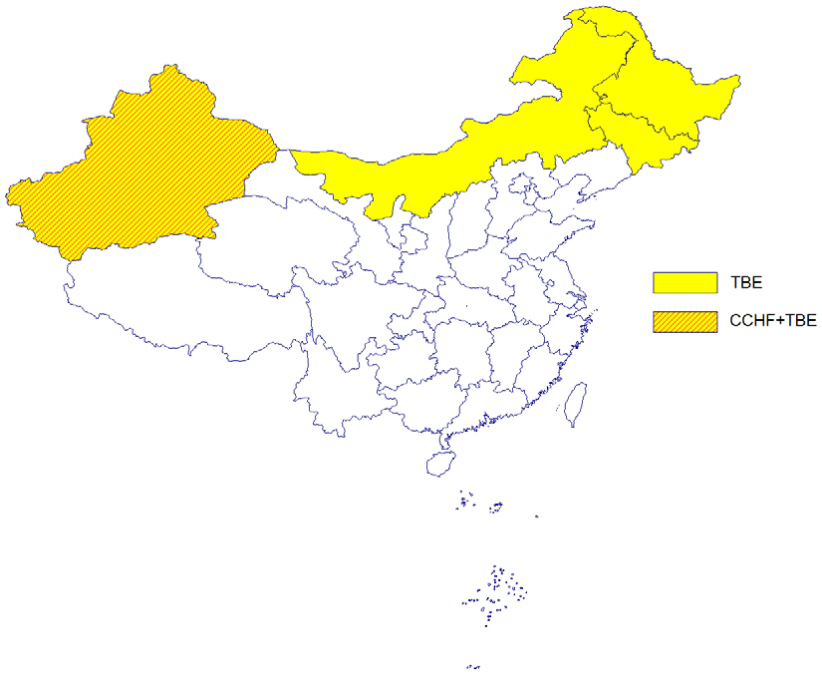
Distribution of tick-borne diseases in mainland China.

To date in China, CCHF cases have only been reported from Xinjiang (Figure 4). However, specific antibodies to CCHFV have been detected in the serum of livestock and humans in areas such as Qinghai province, Inner Mongolia Autonomous Region (hereinafter referred to as Inner Mongolia), Sichuan province, Yunnan province, Hainan province, and Anhui province, which implies that there may be other areas of CCHFV transmission in mainland China [50]. Almost all CCHF cases occur from late March to mid-June with peaks in April and May [50]. Patients are primarily middle-aged male ranchers.

Bachu County in Xinjiang reports most of the CCHF cases in the region, probably because the ecology of the area is semi-desert and is suitable habitat for the vector ticks. From 1965 to 2002, 230 cases were reported from Bachu County with an average annual incidence of 6 [51], [52]. The most serious epidemic occurred in 2001 with 51 cases and 3 deaths reported in Bachu County [52]. Since 2003, no cases have been reported from Xinjiang.

### Prevention and Recommendations

Currently, there is no vaccine available in China to prevent CCHF and the primary prevention is repelling and killing ticks. An education program informing people about preventing tick bites was established in Xinjiang, which may account for there being no cases reported from this area since 2002. Additional active surveillance using sensitive serological methods and molecular biological methods that are currently available [48], [53], [54] are required to better understand the distribution and disease burden caused by this pathogen.

## Tick-Borne Encephalitis

TBEV is tick-borne flavivirus in the family *Flaviviridae*. It is considered one of the most dangerous human infections occurring in Europe and many parts of Asia. TBEV is believed to cause at least 11,000 human cases of encephalitis in Russia and about 3,000 cases in the rest of Europe annually [55]. Tick-borne encephalitis (TBE) has a significant mortality rate depending upon the strain of virus and may cause long-term neurological/neuropsychiatric sequelae [56]. TBE was first described in China in 1943 and TBEV was isolated from patients and ticks in 1952 [57], [58].

### Virus and Vectors

TBEV is classified as one species with three subtypes: the European subtype, the Siberian subtype, and the Far-Eastern subtype [59]. Human infections with the Far-Eastern subtype viruses are usually severe, frequently with encephalitis signs and with a fatality rate of 20% to 40%. In contrast, the Siberian subtype characteristically induces a less severe acute period and a high prevalence of the nonparalytic febrile form of encephalitis; case fatality rates rarely exceed 6% to 8%. Those following infection by European-subtype strains are usually milder, mostly without sequelae; case fatality rates are often as low as 1% to 2% [60].

While *Ixodes ricinus* is the most important vector of the European subtype, *I. persulcatus* is the main vector of the other subtypes. The main vector of TBEV in China is *I. persulcatus*, which is distributed in the forest areas of northeast and northwest China, including Liaoning province, Jilin province, Heilongjiang province, Inner Mongolia, and Xinjiang, particularly in areas characterized as mixed broadleaf-conifer forests.

By far, all TBEV strains isolated in China belong to the Far-Eastern subtype [57], [59]. TBEV strains have been isolated from patients and ticks in north and north-eastern forest areas of China. HLJ-1 strain and Senzhang strain were isolated from brain tissues of a patient from the northeastern forests of China in 1952 and1953, respectively [58], [61]. The Senzhang strain was chosen for the vaccine strain. Also from northeastern forest habitats are the MDJ01 strain (isolated from the sera of a TBE patient) and the “T” and “H” strains (isolated from *I. persulcatus* and brain tissue of an encephalitis patient, respectively) [61], [62]. More recently, six TBE strains (DXAL-5, -12, -13, -16, -18, and -21) have been isolated from *I. persulcatus* collected in forest areas of Heilongjiang province in 2002. Phylogenetic analysis of the E protein gene sequence showed that all six strains belong to the Far-Eastern subtype. The homology of amino acid with Senzhang vaccine strain is higher than 97%, indicating that the vaccine strain likely has good protection against the new isolates [63].

### Epidemiology

The distribution of TBE in China is closely related to the distribution of the tick vectors [64]. There are two foci in mainland China: the Northeast focus and the Xinjiang focus (Figure 4). There is also serological evidence of TBEV in Tibet in western China and in Yunnan located in southwestern China [65].

TBE cases emerge in late April, increase in May, and reach a peak during late May and early June. This period positively correlates with the active season of the tick vector; however, the peak in human cases generally occurs 2 wk following the peak in tick activity [66]. Almost all people infected with TBEV are forest workers, surveyors, rhizotomists, or their family members [66].

### Prevention and Recommendations

A purified virus vaccine derived from the Senzhang strain in 2001 in China has passed stage I, II, and III clinical trials. It has good immunogenicity and notably decreased adverse effect rates compared with inactivated mouse-brain and chicken embryo tissue-derived vaccine developed in 1953 and inactivated chicken embryo cell and hamster kidney cell-derived vaccine developed from 1958 to 1967 [67], [68], [69]. The purified virus vaccine is now produced and marketed in China. Besides vaccination, education programs for preventing tick bites in the forest areas should be implemented in the known epidemic regions.

## Other Arboviruses

Many other arboviruses have been isolated from mosquito and tick vectors, human specimens (serum, CSF, brain tissue), and animal hosts (bats) in mainland China in recent years, including Getah virus (GETV) [70], [71], Chikungunya virus (CHIKV) [72], [73], [74], Sindbis virus (SINDV) [75], [76], Banna virus (BAV) [77], [78], Kyasanur forest disease virus (KFDV) [79], Liaoning virus (LNV) [80], [81], Ross river virus (RRV) [82], Batai virus (BATV) [83], [84], Kadipiro virus (KDV) [85], Tahyna virus (TAHV) [86], and Chaoyang virus (Table 1) [87]. In addition, serosurveys have found evidence of human infection with CHIKV [72], SINDV [75], [88], RRV [82], GETV [71], [89], Semliki Forest virus [89], Kunjun virus [89], Powassan virus [89], Langat virus [89], Sagiyama virus [90], TAHV [86], KFDV [91], [92], and BAV [77], [93]. Many of these viruses have been shown to cause various pathologies ranging from mild fever and arthralgia to encephalitis in humans. Specific evidence is currently lacking to demonstrate the public health impact of these viruses in China.

**Table 1:** Arbovirus isolates and evidence of human infection in mainland China.

Virus (Strain)	Year of First Isolate	Locations Where Isolated	Sources	Human Serology		References
				Antibodies	Location	
GETV (M1)	1964	HN/HeB/YN/SH/GS	Mosquito	c	HN	[70], [71], [89]
CHIKV (B8635)	1986	YN/HN	Mosquito/Bats/Human Serum	b	YN/HN/GD	[72], [73], [74]
SINDV (YN87448)	1987	YN/XJ	Mosquito	b	YN/XJ/FJ	[75], [76], [88]
BAV (Banna_Chinese)	1987	YN/GS/LN/SX/IM/BJ	Mosquito/Pig/Cattle/Human Serum/Human CSF	a,b	YN/HeN/JS/FJ/SD	[77], [78], [93], [95], [96]
KFDV (Nanjianyin)	1989	YN	Human Serum	b	YN/GD/GX/HuB/GZ/HeN/XJ/QH	[79], [91], [92]
RRV (HBb17)	1993	HN	Bat	a,b	HN	[82]
LNV (LNV-NE9712)	1996	JL/XJ	Mosquito	a,b	JL/LN/HLJ	[80], [81]
BATV (YN92-4)	1998	YN	Mosquito	a,b	YN	[83], [84]
KDV (YN0557)	2006	YN	Mosquito	/	/	[85]
TAHV (XJ0625)	2006	XJ/QH/IM	Mosquito	a,b,c	XJ	[86]
Chaoyang Virus (CV)	2008	LN	Mosquito	/	/	[87]

“a” represents IgM antibody positive; “b” represents IgG antibody positive; “c” represents neutralizing antibody positive. HN, Hainan; YN, Yunnan; JL, Jilin; XJ, Xinjiang; HeB, Hebei; SH, Shanghai; GS, Gansu; LN, Liaoning; SX, Shanxi; BJ, Beijing; HeN, Henan; HLJ, Heilongjiang; QH, Qinghai; GD, Guangdong; GX, Guangxi; FJ, Fujian; IM, Inner Mongolia; JS, Jiangsu; GZ, Guizhou; SD, Shandong, HuB, Hubei. “/” means no information available.

However, persistence of encephalitis cases in areas where large-scale JE vaccine programs have been implemented is suggestive of the health burden these arboviral agents may impose [94]. The observations described above underscore the importance of zoonotic arboviruses and reinforce the need to conduct additional investigations into the public health impact of arboviruses and to better understand the epidemiology and ecology of these diseases in mainland China.

## Conclusions

Arboviral diseases, both the well known and relatively obscure, have a great impact on public health in mainland China and have the potential to increase in importance with changes in demographics, land use, and climate change. However, relatively little is known of arboviral epidemiology and transmission ecology in much of the country. It is essential that surveillance be implemented to evaluate and monitor the distribution and true disease burden from arboviral etiologies. In addition, research is needed to better identify and characterize the arboviral strains, vectors, vertebrate hosts, and habitat parameters involved in these complex disease ecologies. Only through accumulation of this additional knowledge will we be able to develop and target appropriate diagnostic services and interventions and to direct resources to developing education programs, vaccines, and therapeutics that may be needed to improve the capacity for infectious disease prevention and control in China and worldwide.

## Supporting Information

Alternative Language Abstract S1 Translation of the abstract into Chinese by XG and GL.
(0.04 MB PDF)
