# Supplementary material for: The Neglected Arboviral Infections in Mainland China
Source: PLoS Negl Trop Dis. 2010 Apr 27;4(4):e624. doi: 10.1371/journal.pntd.0000624 (PMC2860493; doi:10.1371/journal.pntd.0000624)
Supplement: Alternative Language Abstract S1 — Translation of the abstract into Chinese by XG and GL. (0.04 MB PDF) [file pntd.0000624.s001.pdf]

摘要：

虫媒病毒由昆虫媒介传播，可以引起严重的人畜共患疾病，世界各地均有流行，对公共卫生产生巨大影响。中国大陆目前存在并流行四种虫媒病毒病，即乙型脑炎、登革热、克里米亚刚果出血热（又称为新疆出血热）和蜱传脑炎。这四种虫媒病毒病和其他由版纳病毒和 Tahyna 病毒等新分离虫媒病毒引起的虫媒病毒感染给中国大陆带来了很大的公共卫生负担，但是这些疾病或病毒感染并未引起足够的重视。这篇综述收集并汇总了在中国大陆目前流行的 4 种虫媒病毒病的流行病学、主要传播媒介、病原学以及这些疾病的预防和控制等。本文还同时介绍了中国大陆近年来新分离的虫媒病毒及其感染状况等。
